# Resveratrol as a Bioenhancer to Improve Anti-Inflammatory Activities of Apigenin

**DOI:** 10.3390/nu7115485

**Published:** 2015-11-19

**Authors:** Jin-Ah Lee, Sang Keun Ha, EunJung Cho, Inwook Choi

**Affiliations:** Research Group of Nutraceuticals for Metabolic Syndrome, Korea Food Research Institute, 1201-62, Anyangpangyoro, Seongnam, Gyeonggi 463-746, Korea; 07636@kfri.re.kr (J.-A.L.); skha@kfri.re.kr (S.K.H.); Cho.Eun-jung@kfri.re.kr (E.J.C.)

**Keywords:** anti-inflammation, bioenhancer, hepatic metabolites of apigenin, UDP-glucuronosyltransferase (UGT)

## Abstract

The aim of this study was to improve the anti-inflammatory activities of apigenin through co-treatment with resveratrol as a bioenhancer of apigenin. RAW 264.7 cells pretreated with hepatic metabolites formed by the co-metabolism of apigenin and resveratrol (ARMs) in HepG2 cells were stimulated with lipopolysaccharide (LPS). ARMs prominently inhibited (*p* < 0.05) the production of nitric oxide (NO), prostaglandin E_2_ (PGE_2_), interleukin (IL)-1β, IL-6 and TNF-α. Otherwise no such activity was observed by hepatic metabolites of apigenin alone (AMs). ARMs also effectively suppressed protein expressions of inducible nitric oxide synthase (iNOS) and cyclooxygenase-2 (COX-2). Co-administration of apigenin (50 mg/kg) and resveratrol (25 mg/kg) also showed a significant reduction of carrageenan-induced paw edema in mice (61.20% to 23.81%). Co-administration of apigenin and resveratrol led to a 2.39 fold increase in plasma apigenin levels compared to administration of apigenin alone, suggesting that co-administration of resveratrol could increase bioavailability of apigenin. When the action of resveratrol on the main apigenin metabolizing enzymes, UDP-glucuronosyltransferases (UGTs), was investigated, resveratrol mainly inhibited the formation of apigenin glucuronides by UGT1A9 in a non-competitive manner with a *K_i_* value of 7.782 μM. These results suggested that resveratrol helps apigenin to bypass hepatic metabolism and maintain apigenin’s anti-inflammatory activities in the body.

## 1. Introduction

**Figure 1:**
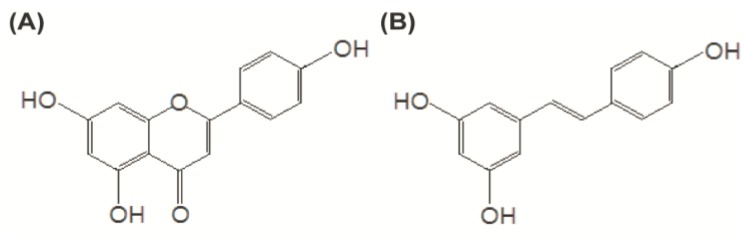
Structures of apigenin (**A**) and resveratrol (**B**).

Apigenin (for chemical structure see Figure 1), a common bioactive flavonoid, is found in high amounts in several herbs including parsley, thyme, and peppermint. It has been reported as an important dietary flavonoid with strong anti-inflammatory activities [1]. It is extensively metabolized and mainly eliminated in humans by first-pass metabolism via glucuronidation and sulfation [2,3]. Rapid metabolism results in large amount of conjugates in the systemic circulation and cause low bioavailability [4]. Bioavailability is the rate and extent to which a therapeutically active substance enters systemic circulation and becomes available at the required site of action. Therefore, enhancement of bioavailability would be of utmost importance in order to exert health effects of flavonoids in a body.

Bioenhancers improve activity and bioavailability of flavonoids in combination therapy. Many studies are increasingly showing interest toward the improvement of bioavailability of a large number of flavonoids by bioenhancers. In the 1920’s, Bose, an acknowledged author of “Pharmacographia India”, reported an enhanced anti-asthmatic effect of an Ayurevdic formula containing *Adhatoda vasica* when administered with long pepper [5]. Piperine, the major plant alkaloid present in *P. nigrum* Linn (Black pepper) and *P. longum* Linn (Long pepper), has bioavailability enhancing activity for curcumin [6] and (-)-epigallocatechin-3-gallate (EGCG) [7]. Another bioenhancer is quercetin that inhibited the activity of UGT1A1 and UGT1A9 [8] and increased bioavailability of EGCG in rats [9].

The main objective of this study is to examine resveratrol as a possible bioenhancer for improving the anti-inflammatory activity of apigenin. To achieve this objective, hepatic metabolites of apigenin in the presence or absence of resveratrol were produced, identified and examined for their suppressive impact on production of pro-inflammatory markers in LPS-stimulated RAW 264.7 cells. In addition, we tried to verify the results obtained from the cell line experiment through an animal study.

## 2. Materials and Methods

### 2.1. Cell Culture

Human hepatoma cell line, HepG2, and murine macrophage cell line, RAW 264.7, were purchased from the American Type Culture Collection (ATCC; Rockville, MD, USA). The cells were incubated in Dulbecco’s modified Eagle’s medium (DMEM; Hyclone, Logan, UT, USA) supplemented with 100 U/mL penicillin, 100 μg/mL streptomycin (Gibco, Grand Island, NY, USA) and 10% fetal bovine serum (FBS; Hyclone, Logan, UT, USA) at 37 °C in a humidified atmosphere of 5% CO_2_.

### 2.2. Production of Hepatic Metabolites of Apigenin and/or Resveratrol in HepG2 Cells

HepG2 cells (2 × 10^6^ cells/well) were seeded into 6 well plates. The cells were incubated with apigenin (20 μM) and/or resveratrol (0, 10, 20, and 40 μM) for 10 h. The medium from HepG2 cells was treated with an equal volume of methanol to precipitate protein and was centrifuged for 2 min at 12,000 rpm. The supernatants were used to test the anti-inflammatory effect after high performance liquid chromatograph (HPLC) analysis.

### 2.3. Determination of NO Production

RAW 264.7 cells (5 × 10^4^ cells/well) were seeded into 96 well plates in phenol red-free DMEM, treated with a standard concentration and hepatic metabolites of apigenin (20 μM) and/or resveratrol (0, 10, 20, and 40 μM) for 30 min, and then incubated in the presence of LPS (1 μg/mL) for 18 h. Nitrite accumulation in the culture medium was measured as an indicator of NO production. Briefly, 50 μL of cell culture medium was mixed with 50 μL of Griess reagent (Sigma, St. Louis, MO, USA). The mixture was incubated at room temperature for 15 min, and the absorbance at 540 nm was measured using a microplate reader (BioTek instruments, Winooski, VT, USA). Fresh culture medium was used as a blank, and the quantity of nitrite was determined by comparison to a sodium nitrite standard curve.

### 2.4. Measurement of PGE_2_ and Pro-Inflammatory Cytokine Production

Cells were treated as described above, and the concentration of PGE_2_, IL-1β, IL-6 and TNF-α in the culture media were quantified using a competitive ELISA kit (PGE_2_, Cayman Chemical, Ann Arbor, MI, USA; IL-1β, IL-6 and TNF-α, R&D system, Minneapolis, MN, USA), according to the manufacturer’s instructions. The absorbance at 450 nm was measured in a microplate reader.

### 2.5. Preparation of Whole Cell Extracts

Cells were treated with hepatic metabolites and standard of apigenin (20 μM) and/or resveratrol (0, 10, 20, and 40 μM) for 30 min, and then incubated in the presence of LPS (1 μg/mL) for 24 h. The cells were then collected by centrifugation and washed twice with phosphate-buffer saline (PBS). Whole cell extracts were prepared using RIPA buffer (Sigma, St. Louis, MO, USA) containing protease inhibitor (Roche, Mannheim, Germany). Protein concentrations were determined using a protein assay reagent (Bio-Rad Laboratories, Hercules, CA, USA) according to the manufacturer’s instructions.

### 2.6. Western Blot Analysis

Equal amounts of total cellular protein (50 μg) were resolved by 10% SDS-polyacrylamide gel electrophoresis (SDS-PAGE) and transferred to a polyvinylidene difluoride (PVDF) membrane. Each immunoblot was incubated with blocking solution (5% skim milk), followed by an overnight incubation at 4 °C with the appropriate primary antibody, as follows: anti-β-actin (1:1000 dilution; Santa Cruz Biotechnology, Santa Cruz, CA, USA); anti-iNOS (1:1000 dilution; BD Bioscience, Bedford, MA, USA); and anti-COX-2 (1:500 dilution; Santa Cruz Biotechnology, Santa Cruz, CA, USA). The blots were washed three times with Tris-buffered saline containing Tween 20 (TBST), and then incubated with a 1:10,000 dilution of horseradish peroxidase (HRP)-conjugated secondary antibody (Santa Cruz Biotechnology, Santa Cruz, CA, USA) for 40 min at room temperature. The blots were again washed four times with TBST, and then developed using an enhanced chemiluminescence (ECL) kit (Pierce Biotechnology, Rockford, IL, USA).

### 2.7. Experimental Animal

Eight weeks old male ICR mice were purchased from Samtako (Osan, Korea). The animals were kept under controlled environmental conditions (22 ± 3 °C with 12/12 h light/dark cycle) for one week prior to the experiment. Animals were given rodent diet and water *ad libitum*. All studies were conducted in accordance with the National Institute of Health “Guide for the Care and Use of Laboratory Animals”.

### 2.8. Carrageenan-Induced Paw Edema in Mice

Edema or swelling is a cardinal sign of acute inflammation and is therefore a useful parameter to look at when testing for agents which may be active in treating acute inflammation. In this experiment, mice were divided into five groups (5 mice per group): control (vehicle), indomethacin (10 mg/kg body weight), apigenin (50 mg/kg body weight), resveratrol (25 mg/kg body weight), and apigenin (50 mg/kg body weight) and resveratrol (25 mg/kg body weight). Vehicle was made by diluting DMSO in 0.5% carboxymethylcellulose (CMC) to give a final concentration of 1% DMSO. All groups with the exception of the indomethacin group were orally administered daily for four days prior to induction of edema. On day 4, all groups were orally administered 3 h prior to induced paw edema. The mice were fasted for 12 h prior to the last administration but with free access to water. Edema was induced by subcutaneous injection of 0.05 mL of PBS containing 1% (*v*/*v*) carrageenan (Sigma, St. Louis, MO, USA) into the right hind paw. Right hind paw volume was measured using a plethysmoneter (Ugo-Basile Co., Varese, Italy). The paw volume was measured at 1 h following carrageenan-induced paw edema in mice. Edema was calculated according to the following equation:

Edema (%) = ((paw volume after carrageenan injection)-(initial paw volume))/(initial paw volume) × 100

The plasma was collected for monitoring the concentration of apigenin in plasma. Blood samples were collected from the eye vein at 5 h after carrageenan injection. Then, the plasma samples were separated by centrifugation at 3000 rpm for 10 min and stored at −70 °C until analysis.

### 2.9. Apigenin and Apigenin Metabolite Analysis

The concentration of apigenin and apigenin-7-glucuronide in medium and plasma was determined by HPLC analysis. The extraction procedure was as follows: 100 μL of plasma was mixed with 10 μL of vitamin C (100 mg/mL). The mixture was vortex mixed for 1 min, and then 600 μL of methanol was added and vortex mixed for 1 min. After centrifugation at 8000 rpm for 10 min, the supernatant was transferred to a clean tube and 20 μL was injected into the HPLC system.

### 2.10. Kinetics of Glucuronidation of Apigenin by UGT1A9

Inhibitory kinetics of resveratrol on glucuronidation of apigenin by recombinant human UGT1A9 (BD, Woburn, MA, USA) was investigated by measuring degrees of glucuronidation of apigenin (20 μM) by UGT1A9 in the presence of resveratrol (10 to 40 μM) dissolved in distilled water. Apigenin and/or resveratrol were preincubated in Tris buffer pH 7.4 (50 mM) with MgCl_2_ (10 mM), alamethicin (25 μg/mL; Sigma, St. Louis, MO, USA) and uridine 5′-diphosphoglucuronic acid (2 mM, UDPGA; Sigma, St. Louis, MO, USA) for 5 min at 37 °C. The reaction was then initiated by addition of recombinant UGT1A9 (5 μg of protein) and incubated for 30 to 360 min at 37 °C. To terminate the reaction, an equal volume of cold methanol was added. The mixture was extracted and subjected to HPLC analysis for determining apigenin and its glucuronide conjugates.

### 2.11. HPLC Analysis

Apigenin and conjugates of apigenin were analyzed by reverse-phase HPLC using an HPLC system (Waters Corp., Milford, MA, USA) with a photodiode array detector (Waters model 2998) and a SunFire^TM^ analytical C18 column (4.6 mm × 150 mm; 5 μm, Waters Corp.). The solvent system was a gradient of solvent A (water:tetrahydrofuran:trifluoroacetic acid, 97.9:2:0.1, *v*/*v*/*v*) and solvent B (acetonitrile): initial composition 17% B; isocratic to 17% B from 0 to 7 min; linear gradient to 25% B from 7 to 15 min; linear gradient to 35% B from 15 to 20 min; linear gradient to 50% B from 20 to 25 min; linear gradient to 100% B from 25 to 30 min; followed by washing and reconditioning the column. The elution was run at a flow rate of 1 mL/min, and the UV spectra were monitored at 350 nm. The injection volume was 20 μL.

### 2.12. Data Analysis

Kinetic parameters (*V_max_*, *K_m_* and *K_i_*) were estimated by nonlinear regression analysis using GraphPad Prism V5 (GraphPad software Inc., San Diego, CA, USA), with models for either Michaelis-Menten or noncompetitive substrate inhibition kinetics. The Michaelis-Menten equation is *V* = *V*_max_ × S/(*K_m_* + S), noncompetitive substrate inhibition equation is *V* = *V*_max_ × S/(*K_m_* + S + S^2^/*K*_i_) where *V* is the velocity of the reaction, S is the substrate concentration, *K_m_* is the Michaelis-Menten constant, *V*_max_ is the maximum velocity and *K_i_* is the substrate inhibition constant.

### 2.13. Statistical Analysis

The data were analyzed using GraphPad Prism V5 (GraphPad software Inc., San Diego, CA, USA). All the data are expressed as mean ± S.E.M. Statistical comparisons between the different treatments were performed using one-way ANOVA with Tukey’s multiple comparison post test.

## 3. Results

### 3.1. Inhibition of NO and PGE_2_ Production, and iNOS and COX-2 Expressions in Vitro

Anti-inflammatory activities of apigenin, hepatic metabolites of apigenin (AMs), and hepatic metabolites of apigenin and resveratrol (ARMs) on LPS-stimulated murine macrophage RAW 264.7 cells were investigated through measuring changes in concentration of inflammatory mediators as well as expression of inflammatory proteins in the cells.

Cell viability was not significantly altered by AMs, ARMs and RMs. So, in the following experiments, cells were treated with AMs, ARMs and RMs (data not shown).

**Figure 2:**
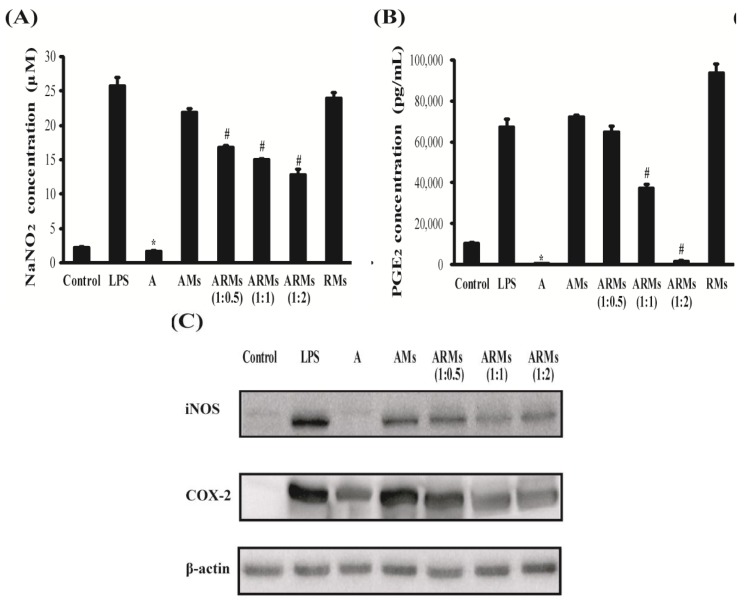
Effects of ARMs on LPS-stimulated NO and PGE_2_ production, and iNOS and COX-2 expressions in RAW264.7 cells. The samples of hepatic metabolites were made as described in the Materials and Methods section. Cells were treated with the standard concentration (A; 20 μM) and hepatic metabolites of apigenin (AMs; 20 μM), hepatic metabolites of apigenin (20 μM) and resveratrol (10, 20 and 40 μM) (ARMs 1:0.5, 1:1 and 1:2), and hepatic metabolites of resveratrol (40 μM) for 30 min, and then incubated in the presence of LPS (1 μg/mL) for 18 h. (**A**) Nitrite accumulation was measured as described in the Materials and Methods section; (**B**) PGE_2_ production was measured as described in the Materials and Methods section. Data were presented as means ± SEM and were representative of triplicate experiments: * *p* < 0.05 compared to cells cultured with LPS (1 μg/mL) alone; # *p* < 0.05 compared to cells cultured with LPS (1 μg/mL) and AMs; (**C**) The expression of iNOS and COX-2 were examined by Western blot analysis.

As shown in Figure 2A and B, addition of apigenin significantly suppressed production of NO and PGE_2_ in LPS-stimulated RAW cells; otherwise addition of AMs and RMs showed no distinctive NO and PGE_2_ suppressing activity indicating that potent anti-inflammatory activities of apigenin might become weakened after metabolization in hepatocytes. When apigenin was co-metabolized with resveratrol (ARMs) and examined for NO and PGE_2_ suppressing activity, however, production of NO and PGE_2_ was suppressed in the order of increased concentrations of resveratrol in ARMs (*p* < 0.05). These results suggested that co-metabolization of resveratrol with apigenin recover to a certain degree anti-inflammatory activity of apigenin after hepatic metabolism.

Western blot analysis showed that ARMs suppressed iNOS and COX-2 expressions; otherwise such a dramatic decrease was not observed by the pretreatment of AMs (Figure 2C).

### 3.2. Inhibition of Secretion of Pro-Inflammatory Cytokines in RAW 264.7 Cells

**Figure 3:**
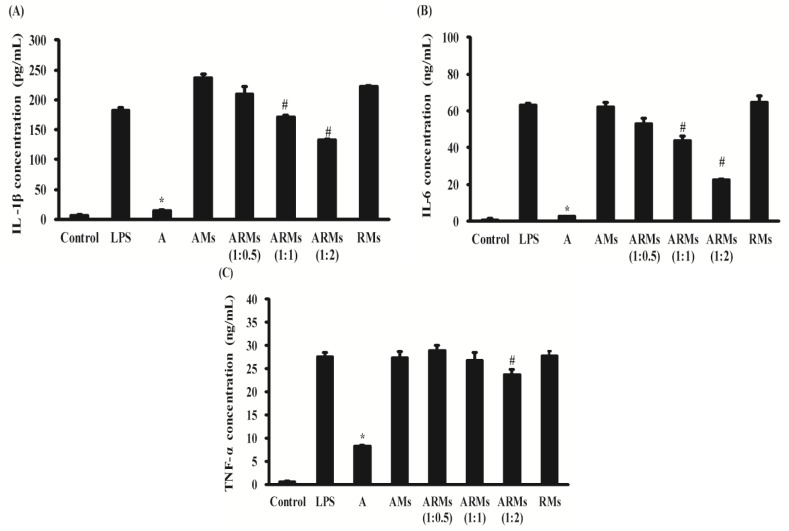
Effects of ARMs on LPS-stimulated IL-1β, IL-6 and TNF-α production in RAW 264.7 cells. The samples of hepatic metabolites were made as described in the Materials and Methods section. Cells were treated with the standard concentration (A; 20 μM) and hepatic metabolites of apigenin (AMs; 20 μM), hepatic metabolites of apigenin (20 μM) and resveratrol (10, 20 and 40 μM) (ARMs 1:0.5, 1:1 and 1:2), and hepatic metabolites of resveratrol (40 μM) for 30 min, and then incubated in the presence of LPS (1 μg/mL) for 18 h. The concentration of (**A**) IL-1β, (**B**) IL-6 and (**C**) TNF-α in the cell culture supernatants were measured as described in the Materials and Methods section. Data were presented as means ± SEM and were representative of triplicate experiments: * *p* < 0.05 compared to cells cultured with LPS (1 μg/mL) alone; # *p* < 0.05 compared to cells cultured with LPS (1 μg/mL) and AMs.

Changes in the supernatant of IL-1β, IL-6 and TNF-α by pretreatments of A, AMs, ARMs, and RMs were measured (Figure 3). As expected, A inhibited the production of IL-1β (14.587 ± 0.636 pg/mL), IL-6 (2.343 ± 0.143 ng/mL) and TNF-α (8.149 ± 0.358 ng/mL) effectively (*p* < 0.05 compared to LPS alone) while AMs did not (236.901 ± 5.386 pg/mL, 61.947 ± 2.562 ng/mL and 27.411 ± 1.272 ng/mL, respectively). RMs also did not inhibit the production of these pro-inflammatory cytokines. ARMs, however, significantly inhibited the production of IL-1β (209.309 ± 12.121 pg/mL to 132.104 ± 1.691 pg/mL; *p* < 0.05 compared to LPS and AMs), IL-6 (52.668 ± 3.173 ng/mL to 22.458 ± 0.554 ng/mL; *p* < 0.05 compared to LPS and AMs) and TNF-α (28.790 ± 1.247 ng/mL to 23.628 ± 1.130 ng/mL; *p* < 0.05 compared to LPS and AMs) in the different concentrations of resveratrol. ARMs were more effective than AMs on suppressing the production of these pro-inflammatory cytokines.

### 3.3. Carrageenan-Induced Paw Edema in Mice

**Figure 4:**
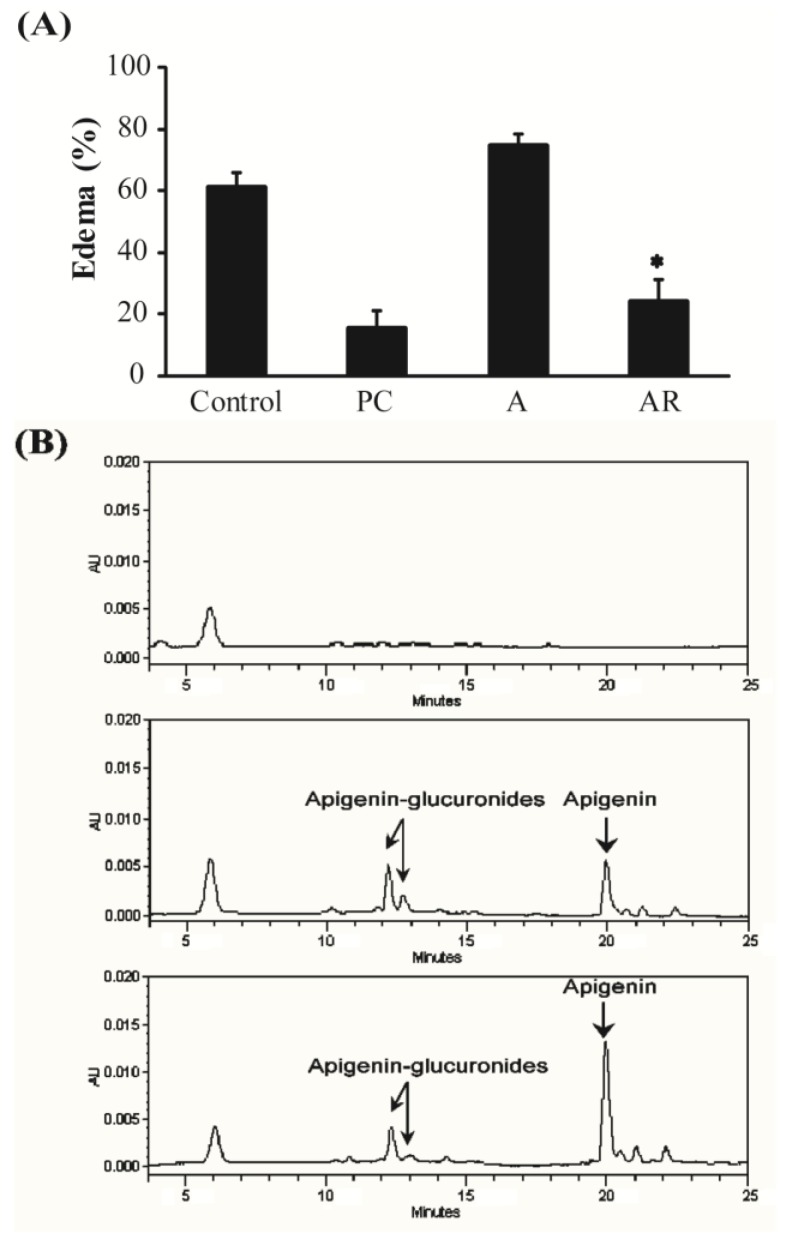
Effects of co-administration of apigenin and resveratrol on carrageenan-induced paw edema in mice. (**A**) Percentage of carrageen-induced paw edema of control group (vehicle), apigenin administered (50 mg/kg body weight), resveratrol administered (25 mg/kg body weight), apigenin and resveratrol administered (50 and 25 mg/kg body weight), and indomethacin (10 mg/kg body weight). Data were expressed as means ± SEM, *n* = 5. * *p* < 0.05 compared to administration of apigenin; (**B**) HPLC chromatograms of plasma of control (up), apigenin administered (middle), and apigenin and resveratrol co-administered (down) groups. Plasma samples were collected and analyzed for apigenin and its metabolites 5 h after carrageenan injection.

We next expanded upon the results of the anti-inflammatory activities from *in vitro* cell line experiments through the use of a carrageenan-induced paw edema mouse model. The increased mouse paw volume after injection of carrageenan indicated development of edema caused by inflammation. As shown in Figure 4A, oral administration of apigenin (50 mg/kg body weight) or resveratrol (25 mg/kg body weight) showed no edema suppressing activity. Co-administration of apigenin and resveratrol (50 and 25 mg/kg body weight, respectively), however, significantly decreased the paw volume down to that of the positive control (indomethacin; 10 mg/kg body weight). After the paw edema experiment, the plasma concentration of apigenin was determined (Figure 4B and Table 1). When apigenin was fed without resveratrol, it was mostly metabolized into its glucuronide conjugates. The plasma concentration of apigenin in the apigenin and resveratrol co-administered group (1084.600 ± 243.508 ng/mL), however, was substantially greater (2.39 fold) than that in the solely apigenin administered group (452.996 ± 155.857 ng/mL). This result indicates that resveratrol may increase absorption of apigenin or/and make apigenin less metabolized by the body, which could result in suppression of paw edema in the mice.

**Table 1:** Mean concentration of apigenin in plasma after oral administration of apigenin and/or resveratrol to mice. Data were represented with mean ± SEM. (*n* = 5).

Group	Concentration of Apigenin (ng/mL)
Apigenin	452.996 ± 155.857
Apigenin + Resveratrol	1084.600 ± 243.508 *

* *p* < 0.01, statistical significance compared with apigenin group.

### 3.4. Kinetic Pattern of Action of Resveratrol on Formation of Glucuronide Conjugates of Apigenin by Recombinant UGT1A9

**Figure 5:**
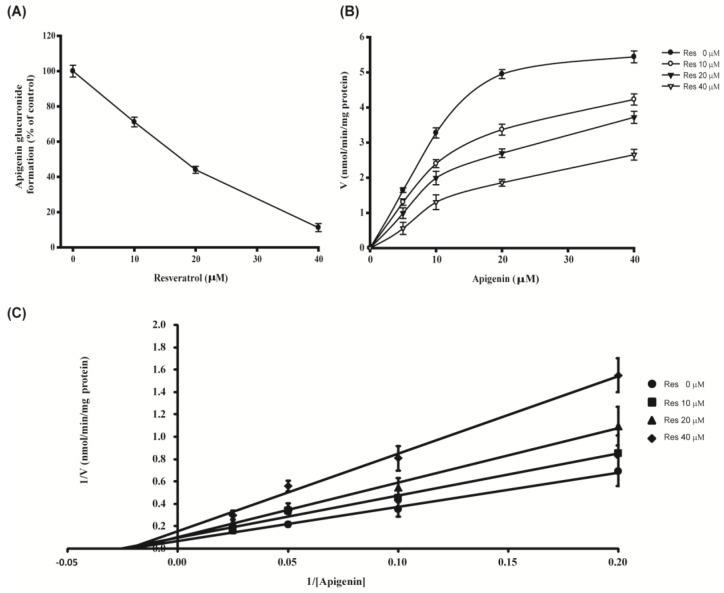
Inhibition of resveratrol towards UGT1A9-catalyzed apigenin glucuronidation (**A**) and kinetic profiles for formation of apigenin glucuronide in the presence of resveratrol by recombinant UGT1A9 (**B** and **C**). Recombinant UGT1A9 (5 μg of protein) was incubated with 5 to 40 μM apigenin and 2 mM UDPGA at 37 °C for 30 to 360 min in the presence (10, 20, and 40 μM) or absence of resveratrol. Data were represented as means ± SEM and were representative of triplicate experiments.

When apigenin was conjugated with glucuronide by various human recombinant UGTs, UGT1A9 was found to be mostly responsible for apigenin glucuronide formation [10]. Therefore, we performed kinetic assays to determine patterns of action of resveratrol on apigenin glucuronide formation by UGT1A9. Resveratrol inhibited production of apigenin glucuronide with a *K_i_* value of 7.782 ± 0.84 μM. The model of inhibition and inhibition kinetic constants of UGT1A9 were determined in the presence of various concentrations of apigenin and resveratrol. The inhibitory pattern of resveratrol on forming apigenin glucuronides by recombinant UGT1A9 was found to be noncompetitive. The *K_m_* for apigenin was 0.478 ± 0.02 μM (Figure 5 and Table 2).

**Table 2:** Kinetic profiles of resveratrol on glucuronide formation from apigenin by recombinant UGT1A9. *V*_max_, *K_m_*, and *K_i_* values were expressed as best-fit values ± SEM of the estimate and were representative of triplicate experiments.

UGT Source	*V*_max_ (nmol/min/mg Protein)	*K_m_* (μM)	*K_i_* (μM)
UGT1A9 ^a^	3.935 ± 0.65	0.478 ± 0.02	7.782 ± 0.84

^a^ Kinetic parameters were calculated by the substrate inhibition equation.

## 4. Discussion

Apigenin has strong anti-inflammatory activity by decreasing IL-1β, IL-6 and TNF-α mRNA levels in LPS-stimulated mouse J774.2 macrophages [11]. Since apigenin is rapidly converted to its glucuronide and sulfate conjugates however, it is difficult to expect such biofunctional activities *in vivo*. Generally, biological activities of conjugated metabolites of flavonoids were considered less effective than their precursors [12,13,14].

To overcome such barriers on effectiveness of flavonoids in a body, bioenhancers that can modulate hepatic metabolism of flavonoids have been introduced. Piperine strongly inhibited the UGT activities [15] and modified the rate of glucuronidation by lowering the endogenous UDP-glucuronic acid content and also by inhibiting the transferase activity [16]. Currently, resveratrol is a dietary chemical under development for its numerous health benefits. It modulates lipid and lipoprotein metabolism [17,18]. Administration of resveratrol in mice resulted in suppressed expression of CYP1A1 [19], and CYP3A4 and CYP2E1 [20] in *in vitro* systems. It inhibits glucuronidation of 7-ethyl-10-hydroxycamptothecin (SN-38), and bilirubin and 7-hydroxytrifluoromethyl coumarin (7-HFC), which is assumed to be mainly catalyzed by UGT1A1 and 1A9 [21,22]. Such modulating activities of resveratrol on metabolizing enzymes in hepatocytes suggest that resveratrol could be used as a bioenhancer for rapidly metabolized flavonoids.

During inflammatory processes, large amounts of pro-inflammatory mediators, including interleukin (IL)-1β, -6, tumor necrosis factor (TNF)-α, and nitric oxide (NO) are secreted [23]. These mediators are generated by iNOS and COX-2 [24,25]. Therefore, selective inhibition of iNOS activity has been established as a therapeutic approach for treating inflammation. Furthermore, COX-2 also is believed to be the isoform responsible for the production of PGE_2_ in various models of inflammation [26]. COX-2 are pro-inflammatory enzymes that are involved in arachidonic acid metabolism and influence biological reactions such as tissue repair and immune response, all of which are associated with inflammation. As observed in our results, apigenin itself was an excellent anti-inflammatory agent. When apigenin was metabolized in hepatocytes, however, its hepatic metabolites did not possess proper anti-inflammatory activities. We observed that ARMs inhibited the protein expression of iNOS and COX-2 and decreased the production of NO and PGE_2_ (Figure 2). This result indicated that NO and PGE_2_ suppression could be mediated via controlling iNOS and COX-2 expression, respectively. NO can promote TNF-α production in mouse macrophages and TNF-α can induce the release of IL-1β and IL-6, which then reciprocally enhances the sensitivity of histiocytes to TNF-α [26]. According to our results, ARMs significantly inhibited the production of IL-1β and IL-6 (Figure 3). TNF-α was inhibited in the ARMs (1:2) treated group. It is reported that apigenin was less potent in regulating TNF-α expression and secretion than IL-1β and IL-6 in the range from 6.25 to 25 μM [27]. Concentrations of apigenin in AMs and ARMs (1:0.5), (1:1), and (1:2) were 3.8 μM, 6.5 μM, 9.0 μM and 12.1 μM, respectively. Concentrations of its major metabolite (apigenin-7-glucuronide) in AMs and ARMs (1:0.5), (1:1), and (1:2) were 13.5 μM, 8.4 μM, 6.5 μM and 2.9. μM, respectively (data not shown). These results suggest that co-treatment of resveratrol could modulate hepatic metabolism of apigenin, resulting in improved anti-inflammatory activities of apigenin.

To verify roles of resveratrol in enhancing anti-inflammatory activities of apigenin, the animal study of paw edema was performed. Carrageenan-induced mice hind paw edema has been widely used for the discovery and evaluation of anti-inflammatory drugs because the relative potency estimates obtained tend to reflect clinical experience. This model allows quantitative assessment of both inflammation and the formation of chemical mediators such as cytokines [28]. As previously observed in *in vitro* experiment, acute inflammation being measured by paw volume was significantly suppressed when apigenin and resveratrol were co-administrated in mice before subcutaneous injection of carrageenan in hind paw (Figure 4). This *in vivo* result further verified the result of *in vitro* experiments.

A difference in bioavailability of apigenin, which was revealed by the different concentrations of apigenin in the medium between AMs and ARMs, will cause differences in their anti-inflammatory activities. These results were further shown by measuring the concentration of apigenin in plasma. After the paw edema experiment, all mice were subjected to an evaluation of the bioavailability of apigenin by measuring apigenin concentrations in plasma. When apigenin was orally administrated in mice, apigenin was rapidly metabolized (Figure 4). Metabolites of apigenin were mostly apigenin-7-*O*-glucuronide (the first peak of apigenin glucuronides on HPLC) when the retention was compared with that of a standard and β-glucuronidase reaction (data not shown). When apigenin was co-administered with resveratrol, however, there was significant increase in concentrations of apigenin in plasma (from 452.996 ± 155.857 ng/mL to 1084.600 ± 243.508 ng/mL) (Figure 4 and Table 1). This result indicates that resveratrol may increase absorption of apigenin or/and inhibit apigenin metabolism in hepatocytes.

UGTs are a superfamily of enzymes responsible for the glucuronidation of numerous endogenous and exogenous small lipophilic compounds, including drugs and xenobiotics; the resulting glucuronide conjugates are generally inactive and water soluble, thus promoting their excretion from the body [29]. Among various UGTs, UGT1A1, 1A3, 1A6, 1A9 and 2B7 have been identified as being responsible for glucuronidation of apigenin [10]. When resveratrol was co-incubated with apigenin in the reaction mixture of UGTs, only glucuronide formation by UGT1A9 was greatly suppressed by resveratrol; otherwise UGT1A1, 1A3 and 1A6 were less affected (data not shown). UGT1A9 has been suggested as the major enzyme contributing to glucuronidation of flavonoids [30]. It is abundant in human liver, but very little occurs in the intestine [31]. Since apigenin was mainly metabolized into glucuronide conjugate by UGT1A9, an effect of resveratrol on inhibiting UGT1A9-mediated glucuronidation of apigenin was determined by kinetic profiles. Kinetic profiles required determination of rates of glucuronidation at various substrate concentrations. The results indicate that resveratrol exerted a typical non-competitive inhibition on UGT1A9-mediated glucuronidation of apigenin with the *K_i_* value of 7.782 ± 0.84 μM and the *K_m_* value of 0.478 ± 0.02 μM (Figure 5 and Table 2).

## 5. Conclusions

We observed that ARMs-induced down-regulation of iNOS and COX-2 expression, and decreased production of NO, PGE_2_, IL-1β and IL-6. This study provides the first evidence of resveratrol modulating hepatic metabolism of apigenin by inhibiting UGT1A9, which, in turn, may result in improved bioavailability of apigenin *in vivo* experiment. These findings suggest that the anti-inflammatory effects of ARMs are mediated by a reduction of the rate of apigenin metabolism by resveratrol, making this flavonoid a potential bioenhancer.
